# Variants in candidate genes and their interactions with smoking on the risk of acute coronary syndrome

**DOI:** 10.7705/biomedica.7379

**Published:** 2025-03-28

**Authors:** Liliana Franco, Natalia Gallego, Cristian Velarde, Diana Valencia, Juan Pablo Pérez-Bedoya, Kelly Betancur, Kelly Marisancen, Paola Parra, Santiago Carvalho, Luisa Parra, Evert Jiménez, Carlos Martínez, Clara Saldarriaga, Juan Carlos Arango, Nathalia González-Jaramillo, Jenny García, Ana Valencia

**Affiliations:** 1. Facultad de Medicina, Universidad Pontificia Bolivariana, Medellín, Colombia Universidad Pontificia Bolivariana Universidad Pontificia Bolivariana Medellín Colombia; 2 Centro de investigaciones, Clínica Cardio VID, Medellín, Colombia Clínica Cardio VID Medellín Colombia; 3 Facultad de Medicina, Universidad de Antioquia, Medellín, Colombia Universidad de Antioquia Universidad de Antioquia Medellín Colombia

**Keywords:** Acute coronary syndrome, genetic association studies, polymorphism, genetic, gene-environment interactions, smoking, case-control studies, síndrome coronario agudo, estudios de asociación genética, polimorfismo genético, interacción gen-ambiente, fumar, estudios de casos y controles

## Abstract

**Introduction.:**

Multiple genetic and environmental factors interact with the development of acute coronary syndrome. Smoking is one of the environmental factors that might alter the metabolic pathways shared by genes associated with this condition.

**Objective.:**

To investigate the association of acute coronary syndrome with genetic variants related to inflammation, lipid metabolism, and platelet aggregation among subjects from the northeastern region of Colombia. The effects of interactions between polymorphisms and smoking were also evaluated.

**Materials and methods.:**

We analyzed data from 330 acute coronary syndrome cases and 430 controls. Associations between 20 polymorphisms and acute coronary syndrome were evaluated using logistic regression, adjusting for confounders. Gene and smoking interaction terms were calculated, and variants were analyzed separately in smokers and non-smokers for their association with acute coronary syndrome.

**Results.:**

Two variants were associated with acute coronary syndrome, rs10455872 in the *LPA* gene (OR = 2.69; 95% CI: 1.61-4.49) and rs429358 in the *APOE* gene (OR = 1.93; 95% CI: 1.30-2.87). We identified smoking interactions with the variants rs6511720 in the *LDLR* gene (p = 0.04) and rs2227631 in the *SERPINE1* gene (p = 0.02), with significant effects in non-smokers (rs6511720: OR = 0.40; 95% CI: 0.19-0.88; and rs2227631: OR = 0.69; 95% CI: 0.48-1.00), but not in smokers (rs6511720: OR = 1.28; 95% CI: 0.66-2.46; and rs2227631: OR = 1.30; 95% CI: 0.91-1.87).

**Conclusions.:**

Variants in the candidate genes *LPA* and *APOE* are associated with an increased risk of acute coronary syndrome in a population from northeastern Colombia. The effects of rs6511720 in *LDLR* and rs2227631 in *SERPINE1* differ according to smoking habits and are significant in non-smokers. These results are helpful for early risk screening of acute coronary syndrome, mainly in individuals without defined conventional risk factors.

## Resumen

**Introducción.:**

Múltiples factores genéticos y ambientales interactúan en el desarrollo del síndrome coronario agudo; el tabaquismo es uno de los factores ambientales que puede alterar los genes implicados en las vías metabólicas asociadas con esta condición.

**Objetivo.:**

Investigar la asociación de variantes genéticas relacionadas con la inflamación, el metabolismo de los lípidos y la agregación plaquetaria, con el riesgo de desarrollar síndrome coronario agudo en personas del nordeste colombiano. Además, evaluar los efectos de la interacción entre los polimorfismos y el hábito de fumar.

**Materiales and métodos.:**

Se analizaron los datos de 330 casos de síndrome coronario agudo y 430 controles. Se evaluó la asociación de 20 polimorfismos con el riesgo de padecer un síndrome coronario agudo mediante regresión logística ajustada por factores de confusión. Se calcularon los términos de la interacción entre las variantes genéticas y el tabaquismo en asociación con el síndrome coronario agudo. Estas asociaciones se evaluaron por separado para fumadores y para no fumadores.

**Resultados.:**

Se encontraron dos variantes asociadas con el síndrome coronario agudo: rs10455872 del gen *LPA* (OR = 2,69; IC _95%_: 1,61-4,49) y rs429358 del gen *APOE* (OR = 1,93; IC _95%_: 1,30-2,87). Se identificaron interacciones entre las variantes genéticas y el tabaquismo para rs6511720 en *LDLR* (p = 0,04) y rs2227631 en *SERPINE1* (p = 0,02). Se observaron efectos estadísticos significativos entre los no fumadores (rs6511720: OR = 0,40; IC _95%_: 0,19-0,88 y rs2227631: OR = 0,69; IC _95%_: 0,48-1,00), pero no en los fumadores (rs6511720: OR = 1,28; IC _95%_: 0,66-2,46 y rs2227631 OR = 1,30; IC _95%_: 0,91-1,87).

**Conclusiones.:**

Las variantes genéticas de *LPA* y *APOE* encontradas en una población del nordeste de Colombia están asociadas con un alto riesgo de desarrollar un síndrome coronario agudo. Los efectos de rs6511720 en *LDLR* y rs2227631 en *SERPINE1* varían entre fumadores y no fumadores, y son estadísticamente significativos en este último grupo. Estos resultados son útiles para el tamizaje temprano del riesgo de padecer el síndrome coronario agudo, principalmente en individuos cuyos factores de riesgo convencionales no son claros.

Cardiovascular diseases are the leading cause of mortality worldwide, accounting for 20.5 million deaths or about one-third of global fatalities in 2021 ^1^. In the Americas, cardiovascular diseases were responsible for two million deaths in 2019 ^2^. Although some Latin American countries show a decreasing trend in mortality rates, in others, the mortality continues to increase compared to more developed regions ^3^.

The acute coronary syndrome is the most important clinical manifestation of cardiovascular diseases. Multiple gene variants have been associated with it and some of its conventional risk factors in different populations ^4^^,^^5^, such as genes involved in inflammatory processes ^6^, lipid metabolism ^7^, coagulation, and platelet aggregation ^8^. However, most gene variants associated with the acute coronary syndrome have a moderate and heterogeneous effect across populations, making it challenging to explain susceptibility to the condition when examined independently ^4^^,^^5^.

A combination of genetic and environmental factors influences the risk of acute coronary syndrome. Therefore, environmental risk factors, genetic variants, gene-gene and gene-environment interactions can provide better predictions for coronary disease risk ^9^.

Smoking contributes significantly to the conventional risk factors associated with acute coronary syndrome. Cigarette smoke promotes a proatherogenic or prothrombotic state through oxidative stress, low-density lipoprotein (LDL) oxidation, cytokine level alteration, platelet aggregation, and prothrombotic and fibrinolytic activation ^10^^,^^11^. Multiple genes modulate the molecular pathways involved in these mechanisms, and the impact of specific genes on the risk of cardiovascular disease and acute coronary syndrome can be modulated by exposure to cigarette smoke ^12^^-^^15^.

Few studies have analyzed the influence of genetic variants and their interaction with environmental factors in the development of acute coronary syndrome or cardiovascular disease in Latin American populations. Since these populations show a history of recent genetic intermixing and particular environmental factors, the association between gene variations and acute coronary syndrome may differ from that of other populations ^16^.

Our objective was to evaluate the effect of genetic variants involved in inflammation, lipid metabolism, and platelet aggregation and their interaction with smoking on the risk of acute coronary syndrome in young subjects from the northeast of Colombia.

## Materials and methods

### Study design and population

We conducted an analytical observational case-control study. Cases were recruited between January 2013 and June 2017 at a reference clinic for cardiovascular diseases in northeastern Colombia. The study included male and female subjects diagnosed for the first time with an acute coronary event at a maximum age of 55 and 65 years, respectively.

Participants had significant coronary stenoses (≥ 50%) in at least one coronary artery. Acute coronary syndrome diagnosis was determined through angiographic and electrocardiographic examinations and serum troponin tests, following the American College of Cardiology and the American Heart Association criteria ^17^.

Controls were healthy individuals from the same population of cases, in the same age range, with no self-reported history of coronary disease or ischemic cerebrovascular events. The control group was selected from a previous study using a probabilistic multistage cluster sampling model stratified by socioeconomic status in Medellín, Colombia ^18^.

Individuals with regular use of cocaine or amphetamines or with a previous diagnosis of immune diseases, thrombophilia, or cancer were excluded from the case and control groups.

### Environmental, sociodemographic, and clinical variables

Environmental exposure and sociodemographic variables were selfreported. Smokers were defined as individuals who had smoked more than 100 cigarettes in their lifetime by the time of the acute coronary syndrome diagnosis ^19^ or their enrollment in the control group. Lipid profile variables were measured using an automatic biochemistry analyzer (VITROS 350™, Ortho Clinical Diagnostics). Diabetes and hypertension data from the control group were obtained through clinical interviews. All clinical variables for the case group were obtained from medical records reviewed by general physicians and reassessed by a cardiologist in case of inconsistencies.

### Gene and polymorphism selection and measuring

We collected 4 ml of peripheral blood from each participant and stored it in tubes containing EDTA anticoagulant. DNA was extracted from leucocytes using the salting-out method ^20^. Samples’ quantity and purity were evaluated by spectrophotometry (Nanodrop model 2000c^TM^, Thermo Fisher, Waltham, USA).

The following criteria were considered for candidate gene and polymorphism selection:

1. Involvement in lipid metabolism, inflammatory responses, platelet aggregation, or vascular remodeling, critical processes in atherosclerosis, and therefore, acute coronary syndrome;
2. Consistent association with acute coronary syndrome across different populations, and
3. Minor allele frequency ≥ 5%, according to Ensembl reports for Colombian populations ^21^ (supplementary table 1).

Additionally, to control potential effects of the population genetic structure, the percentage of individual genetic admixture was estimated through genotyping a panel of 10 ancestry-informative markers (supplementary table 2). These estimations were obtained using the statistical software Structure, version 2.3.4 ^22^. We calculated the genome proportions corresponding to each of the three major ancestral components of the Latin American population: European, Amerindian, and African. Since the predominant ancestry for the studied population -Antioquia- is European ^23^ and is collinear with the others (less represented ancestries), we included only European ancestry as a covariable in the multivariate association analysis.

Single nucleotide polymorphisms (SNP) genotyping was performed using the polymerase chain reaction (PCR) method. Samples were amplified with TaqMan™ probes in an Applied Biosystems 7500 Fast Real-Time PCR System™ (California, USA) at the Genetic Resources Core Facility of Johns Hopkins University, Baltimore, USA. Ancestry-informative marker, insertion/ deletion polymorphism, and Alu element typification were performed through conventional PCR and electrophoresis in 2 to 3% agarose gels, depending on the differences in alleles’ base pairs.

Typification was performed without knowing the case or control status. Two researchers evaluated genotypes independently. In case of discrepancies, readings were replicated until reaching a consensus. If the discrepancies persisted, such genotypes were excluded from the analysis. Five percent of each polymorphism typed was repeated in randomly selected samples. Markers showing a discrepancy rate higher than 5% between replicates, a typification rate lower than 95%, and a significant deviation from the Hardy- Weinberg equilibrium in the control group were excluded from the analysis (p < 0.05).

### Statistical analysis

Categorical variables were compared between cases and controls by applying a chi-square test, while quantitative variables were analyzed with a Mann-Whitney U test. The association of each genetic variant with acute coronary syndrome was evaluated using logistic regression analysis, adjusted for age, sex, education level, European ethnicity, diabetes, hypertension, and dyslipidemia. These confounders were selected based on their clinical and epidemiological relevance to acute coronary syndrome. We assessed allelic and additive models. Odds ratios (OR) and their 95% confidence intervals (CI) were estimated by referencing the category with the most individuals; p values lower than 0.003 were considered significant (as corrected for 15 independent tests, equivalent to the number of genes evaluated).

The corresponding interaction term was included in the regression model to assess gene-smoking interactions. When the p value was less than 0.05, the models were stratified by smoking status (smokers versus non-smokers).

We estimated the most probable haplotypes composed of the variants using the D’ coefficient to assess whether the combination of variants within a single gene showed a more significant effect. We used the software package PLINK, version 1.9 ^24^.

All procedures included in our study were approved by the Ethics Committee at the *Universidad Pontificia Bolivariana* (Minute No. 8 of 2012) and were conducted according to the standards of the Declaration of Helsinki. Informed consent was obtained from all subjects before their inclusion in the study.

## Results

A total of 760 individuals were enrolled, 330 cases and 430 controls. After assessing the Hardy-Weinberg equilibrium and applying quality filters, 20 polymorphisms in 15 genes were evaluated (supplementary table 3).

Traditional risk factors for acute coronary syndrome, such as male gender, age, diabetes, hypertension, dyslipidemia, and smoking habits, were significantly more common in cases compared to controls (table 1). HDL was at lower levels among cases, and acute coronary syndrome cases had a higher education level and higher percentages of European ancestry than the control group (table 1). Smoking was more common in acute coronary syndrome cases and increased the risk of acute coronary syndrome (OR = 3.51; 95% CI: 2.57-4.79).

**Table 1:** Baseline characteristics of acute coronary syndrome cases and controls

Characteristics		Acute coronary syndrome (N = 330)		Controls (N = 430)		p value
Agea (years)						
	Male	50	(46-53)	50	(43.5-56)	0.58
	Female	54	(50-59)	50	(45-56)	< 0.001
Sex^b^						
	Male [n (%)]	218	(66.1)	156	(36.3)	< 0.001
Comorbidities^b^ [n (%)]						
	Diabetes mellitus		(18.6)	18	(4.2)	< 0.001
	Hypertension		(47.8)	94	(21.9)	< 0.001
	Dyslipidemia	264	(80.5)	309	(71.9)	0.01
Lipid profile^a^ (mg/dl)						
	Cholesterol	193	(163-221)	198	(174-229)	0.02
	Triglycerides	169	(123-244)	165	(120-233)	0.35
	HDL	33	(28.3-40)	42	(35-50)	< 0.001
	LDL	117	(94-193)	116	(95-140)	0.72
Smoking^b^ [n (%)]						
	Non-smoker	132	(40.0)	300	(69.9)	
	Smoker	198	(60.0)	129	(30.1)	< 0.001
Education (years)^b^ [n (%)]						
	≤ 6	76	(25.0)	193	(45.4)	
	7-12	151	(49.7)	167	(39.3)	< 0.001
	> 12	77	(25.3)	65	(15.3)	
Socioeconomic status^b^ [n (%)]						
	Low	138	(45.7)	211	(49.4)	
	Medium	139	(46.0)	198	(46.4)	0.06
	High	25	(8.3)	18	(4.2)	
Ancestry^a^ (%)						
	European	89.6	(80.0-93.6)	87.1	(73.5-93.1)	0.03
	Amerindian	4.4	(2.7-8.7)	5.1	(2.8-10.9)	0.30
	African	4.6	(2.6-8.7)	4.9	(2.8-9.8)	0.12

OR: Odds ratio; CI: Confidence interval

^a^ Quantitative variables are shown as medians and interquartile ranges.

^b^ Qualitative variables are shown as absolute and percentual distribution.

### Genetic association analysis

Two variants showed significant differences in allelic and genotypic distribution between acute coronary syndrome cases and controls after adjusting for confounders (table 2, complete results in supplementary table 4). Among the variants involved in lipid metabolism, we identified a significant association between acute coronary syndrome and the G allele of rs10455872 in the LPA gene (OR = 2.69; 95% CI: 1.61-4.49). Also, the C allele of rs429358 in the APOE gene was associated with a higher susceptibility to developing acute coronary syndrome (OR = 1.93; 95% CI: 1.30-2.87). In both variants, the number of homozygous individuals with the risk allele was limited, preventing an evaluation of the effects of this genotype. Nevertheless, the heterozygous genotype showed a significant association (table 2).

**Table 2:** Allele and genotype distribution in acute coronary syndrome patients and healthy controls

Gen (SNP)	Alleles and genotypes	Acute coronary syndrome n (%)	Controls OR (95% CI) n (%) Raw	p value OR (95% CI) Adjusted^a^	p value
*LPA* (rs10455872)	G	65 (10.0)	32 (3.9) 2.78 (1.80-4.30)	< 0.001 2.69 (1.61-4.49)	< 0.001
	G/G	3 (0.9)	0 (0.0) NE	NE	
	G/A	59 (18.2)	32 (7.7) 2.69 (1.70-4.26)	< 0.001 2.39 (1.38-4.15)	0.002
	A/A	263 (80.9)	384 (92.3) 1	1	
*APOE* (rs429358)	C	87 (13.5)	72 (8.6) 1.69 (1.21-2.35)	0.002 1.93 (1.30-2.87)	0.001
	C/C	4 (1.3)	3 (0.7) NE	NE	
	C/T	79 (24.9)	66 (15.8) 1.79 (1.24-2.58)	0.002 2.04 (1.29-3.24)	0.002
	T/T	234 (73.80)	349 (83.5) 1	1	

SNP: single nucleotide polymorphism; OR: odds ratio; CI: confidence interval; NE: non-estimated

^a^ Odds ratios were adjusted by age, gender, education level, European ethnicity, diabetes, hypertension, and smoking.

Other variants in the genes *SORT1, ABCA1, APOE, APOA1, CXCL12*, and *IFNG* showed significant confidence intervals but did not reach statistical significance after multiple testing corrections (supplementary table 4).

Haplotypes formed by the variants that exhibited linkage disequilibrium are displayed in supplementary table 5 and had a comparable effect to the variants regarded individually.

### Interactions between genetic variants and smoking habits on the development of acute coronary syndrome

Among variants involved in lipid metabolism, a statistically significant interaction was found between smoking and rs651120 (*LDLR*) in the allelic and genotypic models. The T allele had a protective effect on acute coronary syndrome in non-smokers (OR = 0.40; 95% CI: 0.19-0.88), while in smokers, the same association was not detected (OR = 1.28; 95% CI: 0.66-2.46) (table 3).

**Table 3:** Effect of *LDLR* and *SERPINE1* on acute coronary syndrome, stratified by smoking status

Gen (SNP)	Alleles and genotypes	Smokers (N = 327)				Non-smokers (N = 431)				Interaction (p value)
		Acute coronary syndrome n (%)	Controls n (%)	OR (95% CI) Adjusted^a^	p value	Acute coronary syndrome n (%)	Controls n (%)	OR (95% CI) Adjusted^a^	p value		T	41 (7.9)	68 (7.1)	1.28 (0.66-2.46)	0.47	10 (3.8)	50 (8.5)	0.40 (0.19-0.88)	0.02	0.04
*LDLR* (rs6511720)
	T/T	1 (0.5)	2 (1.6)	NE		0 (0)	1 (0.3)	NE		
	G/T	29 (14.7)	14 (11.0)	1.61 (0.74-3.05)	0.23	10 (7.6)	48 (16.3)	0.44 (0.20-0.99)	0.05	0.03
	G/G	167 (87.8)	111 (87.4)	1		121 (92.4)	245 (83.3)	1			A	153 (38.6)	89 (34.5)	1.30 (0.91-1.87)	0.15	75 (28.6)	217 (36.2)	0.69 (0.48-1.00)	0.05	0.02
*SERPINE1* (rs2227631)
	A/A	32 (16.2)	15 (11.6)	1.70 (0.76-3.84)	0.20	11 (8.4)	38 (12.7)	0.64 (0.28-1.49)	0.36	0.10
	A/G	89 (45.0)	59 (45.7)	0.99 (0.57-1.72)	0.96	53 (40.5)	141 (47.0)	0.55 (0.32-0.96)	0.03	0.17
	G/G	77 (38.9)	55 (42.6)	1		67 (51.1)	121 (40.3)	1		

SNP: single nucleotide polymorphism; OR: odds ratio; CI: confidence interval; NE: non-estimated

^a^ Odds ratios were adjusted by age, gender, education level, European ethnicity, diabetes, and hypertension. For variant rs2227631, odds ratios were also adjusted by dyslipidemia.

Variant rs2227631 (*SERPINE1*), involved in platelet aggregation, showed different effects according to smoking status. An allele had a protective effect in non-smokers (OR = 0.69; 95% CI: 0.48- 1.00) but lacked statistical significance in smokers (OR = 1.30; 95% CI: 0.91-1.87) (table 3).

## Discussion

Polymorphisms in the *LPA* and *APOE* genes were significantly associated with early acute coronary syndrome. The magnitude of these effects is similar to those reported in other ethnic groups and had no interaction with smoking status. We observed a robust association between the G allele of rs10455872 in the *LPA* gene and the increased risk of acute coronary syndrome. This allele is associated with increased lipoprotein A levels, a preferential carrier of pro-inflammatory oxidized phospholipids ^25^. It also contributes to increased TNF-alpha cytokine levels, endothelial cells’ downregulation of plasminogen activators, and the balance between thrombi formation and fibrinolysis ^26^^-^^28^. All these factors play an important role in the development of acute coronary syndrome, and Mendelian randomization studies support the causal relationship between lipoprotein A and cardiovascular diseases ^27^.

A significant association was found between the C allele of rs429358 in the *APOE* gene and the increased risk of acute coronary syndrome. The C allele in both rs429358 and rs7412 polymorphisms forms the Apo ε4 haplotype, which has consistently shown an atherogenic effect and has been associated with an increased risk of myocardial infarction ^29^. This is the first time this effect has been reported in a Latin American population. In contrast with previous reports on the Caucasian population, we did not find an interaction between variant rs429358 and smoking habits regarding the risk of acute coronary syndrome in our population ^13^. This polymorphism affects lipid metabolism and cytokine levels ^28^^,^^30^, contributing to acute coronary syndrome development.

To our knowledge, this report is the first to indicate that smoking habits modify the protective effect of genetic variants in the *LDLR* and *SERPINE1* genes regarding acute coronary syndrome.

*LDLR* gene encodes a receptor primarily expressed in the liver, responsible for removing low-density lipoproteins (LDL) from the bloodstream. We found that the T allele of the variant rs6511720 confers a protective effect against the risk of acute coronary syndrome only in non-smokers. The intron variant rs6511720 has been associated with plasma levels of LDL and risk of acute coronary syndrome ^5^. Reports by Fairoozy and colleagues also support our results, as they demonstrated that transcription factors preferentially bind to allele T of rs6511720, upregulating the expression of *LDLR*^31^. Although the interaction between variants in the LDLR gene and smoking in humans has yet to be studied, Ma and colleagues found that mice exposed to cigarette smoke exhibited significantly higher levels of total cholesterol, triglycerides, and LDL, along with lower levels of HDL-C, compared to the controls. Furthermore, this exposure significantly reduced the expression of LDLR receptors in the liver ^11^.

We also identified a gene-smoking interaction involving the *SERPINE1* gene. The A allele of rs2227631 appears to confer protection against acute coronary syndrome among non-smokers. This protective effect against acute coronary syndrome risk was reported among the Mexican population, with admixture components similar to those of the Colombian population. However, such a study did not evaluate the interaction with smoking ^32^. Some studies conducted among the Caucasian and Asian populations have reported an interaction between smoking and haplotypes including the promotor variants rs2227631 and the insertion/deletion -675 4G/5G (rs1799762), both in high linkage disequilibrium.

In contrast to our findings, the haplotype containing the A allele of rs2227631 and the G allele of rs1799762-4 significantly increased the risk of coronary heart disease only in non-smokers ^12^. The gene *SERPINE1* encodes the endothelial plasminogen activator inhibitor 1 (PAI-1). Increased PAI-1 plasma levels lead to reduced fibrinolysis and thrombi formation and, consequently, a higher risk of acute coronary syndrome events ^33^. The A allele of the rs2227631 polymorphism is associated with higher levels of PAI-1 in plasma ^34^, and the G allele of rs1799762-5 has been reported to contain a binding site for the suppressor protein that reduces PAI-1 transcription, which would explain lower levels of this protein in plasma ^35^. Additionally, disagreement remains on whether the association of variant rs2227631 with PAI-1 levels is a direct relation or the consequence of its strong linkage disequilibrium with the insertion/deletion variant rs1799762 ^12^^,^^34^^,^^36^. Linkage disequilibrium patterns vary among populations, which can alter the risk or protective alleles among ethnic groups.

The interaction between the genetic variants identified here and smoking may result in the accelerated formation of atheromatous plaques caused by cigarette smoke. This effect occurs through multiple pathways, including alterations of lipid metabolism, increased levels of oxidized LDL (uptake mainly by macrophages), alterations of thrombotic and fibrinolytic factors, and platelet-mediated pathways ^11^^,^^37^ involving proteins encoded by *LDLR* and *SERPINE1* genes. These findings suggest that some individuals in our population may have genetic factors serving as key determinants in the development of atheromatous plaques, even in the absence of a major conventional risk factor like smoking.

This study has some limitations. First, it was not possible to measure all the variables previously associated with acute coronary syndrome and which could play an essential role in the causality model, *e.g*., obesity, sedentarism, and alcohol consumption. Nevertheless, the statistical adjustment -based on other related variables- reduced the residual confounding, and the estimated effects may represent the actual population. Additionally, some interactions might not have been identified since our sample size had 80% power to find interaction effects equal to 1.9, which is relatively high. Therefore, this study lacks enough power to detect risk factors and interactions with smaller effects. Finally, the ethnicity estimation was based on information from only ten ancestry-informative markers ^38^, which may lead to an inaccurate estimate. Previous studies in the Colombian population found that ethnicity strongly correlates with socioeconomic status, which suggests that non-genetic factors related to ethnicity may partially explain associations with the risk of complex diseases ^39^. Considering this, we expect to have corrected, at least partially, the confounding generated by risk factors related to ethnic origins when we adjusted the models for other variables associated with ancestry, such as education level. In addition, a sensitivity analysis showed similar results without adjusting for ancestry.

This study contributes to the evidence on acute coronary syndrome genetic etiology in the Latin American population and its interaction with smoking. We identified a highly significant association of genetic variants in the *LPA* and *APOE* genes with early acute coronary syndrome. Additionally, smoking modifies the effect of variants in the *LDLR* and *SERPINE1* genes by inhibiting the protective alleles in the smoker group. These observations highlight the importance of gene-environment interaction on the risk of acute coronary syndrome and the need to consider such effects in genetic studies.

Our findings suggest that distinct risk groups should be established, as certain genetic factors play a crucial role for individuals who develop acute coronary syndrome in the absence of conventional risk factors. In contrast, others are relevant, for example, lifestyle factors such as cigarette smoke. Such an approach could help design risk assessment scales in the future to aid in early diagnosis and prevent cardiovascular events.

Furthermore, it is essential to validate genetic risk scores accounting for interactions between genetic and conventional risk factors. This will help improve the early diagnosis of acute coronary syndrome and focus on preventive strategies for the higher-risk populations, ultimately reducing the social and economic burden imposed by cardiovascular diseases.

## Supplementary files

**Supplementary table 1.** Candidate genes and genetic variables evaluated

Function	Gen	Chromosome	SNP	Variation type	Minor	frequency allele^a^
Lipid metabolism	*PCSK9*	1p32.3	rs11206510	Intergenic	C	0.13
	*SORT1*	1p13.3	rs646776	Downstream	C	0.21
			rs599839	Downstream	G	0.25
	*LPA*	6q25.3	rs3798220	I [Ile] - ⇒ M [Met]	C	0.18
			rs10455872	Intronic	G	0.06
	*ABCA1*	9q31.1	rs2230806	R [Arg] ⇒ K [Lys]	T	0.34
	*ZPR1 - APOA5*	11q23.3	rs964184	3' UTR	G	0.25
	*APOE*	19q13.2	rs429358	C [Cys] ⇒ R [Arg]	C	0.15
			rs7412	R [Arg] ⇒ C [Cys]	T	0.09
Inflammation	*APOE -APOC1*	19q13.32	rs4420638	Downstream	G	0.10
	*LDLR*	19p13.1-p13.3	rs6511720	Intronic	T	0.11
	*IL10*	1q31-q32	rs1800896	Upstream	C	0.34
			rs1800872	Upstream	T	0.32
	*TNF*	6p21.3	rs1800630	Upstream	A	0.12
Platelet aggregation			rs1799964	Upstream	C	0.18
	*IFNG*	12q14	rs1861493	Intronic	G	0.22
	*CXCL12*	10q11.2	rs501120	Downstream	C	0.21
			rs1746048	Regulator region	T	0.23
	*ABO*	9q32.2	rs579459	Upstream	C	0.13
	*ITGB3*	17q21.32	rs5918	L [Leu] ⇒ P [Pro]	C	0.11
			rs4642	Synonym	A	0.32
	*SERPINE1*	7q21.3-q22	rs2227631	Upstream	A	0.45

SNP: single nucleotide polymorphism

^a^ Frequencies from the population of Medellín (Colombia), reported in the Ensembl genome browser (20)

**Supplementary table 2.** Ancestry informative markers

Marker (variant)	Genome position hg19	Type	Allele frequency of the insertion or the derived allele in the ancestral populations^a^			HWE
			African	European	Amerindian	p value
PEAR1 (rs12041331)	chr1:156869714	SNP	0.54	0.94	0.61	0.05
MID1469 (rs2307665)	chr2:150239284	INDEL	0.77	0.37	0.94	0.365
Ya5ACA1153	chr4:181549441-181549742	ALU	0.31	0.27	0.83	0.485
MID944 (rs1611027)	chr5:92314547-92314576	INDEL	0.89	0.39	0.95	0.175
MID2062 (rs2308254)	chr6:40086897	INDEL	0.42	0.30	0.93	0.47
PON1 (rs662)	chr7:94937446	SNP	0.22	0.69	0.41	0.82
Ya5_541	chr10:35593223-35593539	ALU	0.06	0.55	0.11	0.49
CYP2C19 (rs12248560)	chr10:96521657	SNP	0.25	0.22	0.02	0.47
Ya5ACE (rs4646994)	chr17:61565901-61565902	ALU	0.35	0.35	0.72	0.24
MID154 (rs16434)	chr20:32677028-32677053	INDEL	0.82	0.26	0.15	0.18

HWE: Hardy-Weinberg equilibrium; Chr: chromosome; SNP: single nucleotide polymorphism; INDEL: insertion/deletion; ALU: transposons from the short interspersed nuclear elements family

^a^ Reference populations from the Ensembl genome browser; African: Yoruba; European: CEU (Europeans from Utah); Amerindian: CHB (Han Chinese from Beijing) (20)

**Supplementary table 3.** Hardy-Weinberg equilibrium test

Gen	Chromosome	SNP	Group	Allele 1 (A1)	Allele 2 (A2)	Genotypic frequencies	Heterozygote’s frequencies		p value
						A1A1/A1A2/A2A2	Observed	Expected	
*PCSK9*	1	rs11206510	Cases	C	T	5/63/258	0.19	0.20	0.58
			Controls	C	T	4/86/328	0.21	0.20	0.81
*SORT1*	1	rs646776	Cases	C	T	18/95/217	0.29	0.32	0.09
			Controls	C	T	27/147/256	0.34	0.36	0.35
		rs599839	Cases	G	A	28/100/193	0.31	0.37	0.01
			Controls	G	A	38/168/208	0.41	0.42	0.64
*IL10*	1	rs1800872	Cases	T	G	30/130/170	0.39	0.41	0.50
			Controls	T	G	44/169/217	0.39	0.42	0.21
		rs1800896	Cases	C	T	36/139/151	0.43	0.44	0.62
			Controls	C	T	42/204/173	0.49	0.45	0.13
*TNF*	6	rs1799964	Cases	C	T	16/97/207	0.30	0.32	0.30
			Controls	C	T	22/147/249	0.35	0.35	1.00
		rs1800630	Cases	A	C	22/60/212	0.2	0.29	< 0.01
			Controls	A	C	35/82/248	0.22	0.33	< 0.01
*LPA*	6	rs3798220	Cases	C	T	7/78/244	0.24	0.24	0.82
			Controls	C	T	13/99/318	0.23	0.25	0.12
		rs10455872	Cases	G	A	3/59/263	0.18	0.18	1
			Controls	G	A	0/32/384	0.08	0.07	1
*SERPINE1*	7	rs2227631	Cases	A	G	43/142/144	0.43	0.45	0.40
			Controls	A	G	53/200/177	0.47	0.46	0.83
*ABCA1*	9	rs2230806	Cases	T	C	44/138/145	0.42	0.45	0.22
			Controls	T	C	64/198/159	0.47	0.48	0.84
*ABO*	9	rs579459	Cases	C	T	10/110/207	0.34	0.32	0.39
			Controls	C	T	15/104/303	0.25	0.27	0.14
*CXCL12*	10	rs501120	Cases	C	T	13/118/198	0.36	0.34	0.42
			Controls	C	T	35/169/226	0.39	0.40	0.72
		rs1746048	Cases	T	C	12/114/199	0.35	0.34	0.51
			Controls	T	C	36/163/220	0.39	0.40	0.47
*ZPR1, APOA5-A4-C3-A1*	11	rs964184	Cases	G	C	41/126/158	0.39	0.44	0.06
			Controls	G	C	37/149/231	0.36	0.39	0.08
*IFNG*	12	rs1861493	Cases	G	A	9/134/180	0.42	0.36	0.01
			Controls	G	A	21/144/254	0.34	0.34	0.89
*ITGB3*	17	rs5918	Cases	C	T	5/68/256	0.21	0.21	0.79
			Controls	C	T	6/92/331	0.22	0.21	1
		rs4642a	Cases	G	A	38/120/145	0.40	0.44	0.11
			Controls	G	A	44/156/198	0.39	0.43	0.13
*LDLR*	19	rs6511720	Cases	T	G	1/39/288	0.12	0.12	1
			Controls	T	G	3/62/357	0.15	0.15	0.74
*APOE*	19	rs429358	Cases	C	T	4/79/234	0.25	0.24	0.48
			Controls	C	T	3/66/349	0.16	0.16	1
		rs7412	Cases	T	C	3/26/292	0.08	0.10	0.03
			Controls	T	C	1/61/359	0.15	0.15	0.50
*APOE-APOC1*	19	rs4420638	Cases	G	A	0/70/257	0.21	0.19	0.04
			Controls	G	A	0/57/365	0.14	0.13	0.24

SNP: Single nucleotide polymorphism.

^a^ Variant with typification percentage less than 95%

**Supplementary table 4.** Allele and genotype distribution in acute coronary syndrome patients and healthy controls of all polymorphisms evaluated

Gen (SNP)	Alleles and genotypes	Acute coronary syndrome n (%)	Controls n (%)	OR (95% IC) raw	p value	OR (95% IC) Adjusted^a^	p value
*PCSK9* *rs11206510*	C	73 (11.2)	94 (11.2)	1.00 (0.72-1.38)	0.98	1.15 (0.78-1.70)	0.47
	C/C	5 (1.5)	4 (1)	NE		NE	
	T/C	63 (19.3)	86 (20.6)	0.93 (0.65-1.34)	0.70	0.93 (0.59-1.48)	0.77
	T/T	258 (79.1)	328 (78.5)	1		1	
*SORT1* *rs646776*	C	131 (20.0)	201 (23.4)	0.81 (0.63 -1.04)	0.10	0.67 (0.59-0.89)	0.01
	C/C	18 (5.5)	27 (6.3)	0.79 (0.42-1.47)	0.45	0.67 (0.31-1.44)	0.30
	T/C	95 (28.8)	147 (34.2)	0.76 (0.56 -1.05)	0.09	0.62 (0.42-0.92)	0.02
	T/T	217 (65.8)	256 (59.5)	1		1	
*SORT1* *rs599839*	G	156 (24.3)	245 (29.5)	0.77 (0.61-0.97)	0.03	0.70 (0.54-0.92)	0.01
	G/G	28 (8.7)	38 (9.2)	0.79 (0.47-1.34)	0.39	0.80 (0.42-1.52)	0.49
	G/A	100 (31.2)	168 (40.6)	0.64 (0.47-0.88)	0.01	0.58 (0. 39-0.87)	0.01
	A/A	193 (60.1)	208 (50.2)	1		1	
*LPA* *rs3798220*	C	92 (14.0)	125 (14.5)	0.96 (0.72-1.28)	0.76	1.00 (0.71-1.42)	0.98
	C/C	7 (2.1)	13 (3)	0.70 (0.28-1.79)	0.88	0.78 (0.22-2.73)	0.68
	T/C	78 (23.7)	99 (23.0)	1.03 (0.73-1.44)	0.46	1.18 (0.77-1.80)	0.46
	T/T	244 (74.2)	318 (74.0)	1		1	
*LPA* *rs10455872*	G	65 (10.0)	32 (3.9)	2.78 (1.80-4.30)	<0.001	2.69 (1.61-4.49)	<0.001
	G/G	3 (0.9)	0 (0.0)	NE		NE	
	G/A	59 (18.2)	32 (7.7)	2.69 (1.70-4.26)	<0.001	2.39 (1.38-4.15)	0.002
	A/A	263 (80.9)	384 (92.3)	1		1	
*ABCA1* *rs2230806*	T	226 (34.6)	326 (38.7)	0.84 (0.68-1.03)	0.10	0.88 (0.69-1.13)	0.31
	T/T	44 (13.5)	64 (15.2)	0.75 (0.48-1.18)	0.21	0.79 (0.45-1.38)	0.40
	C/T	138 (42.2)	198 (47.0)	0.76 (0.56-1.05)	0.09	0.65 (0.44-0.96)	0.03
	C/C	145 (44.3)	159 (37.8)	1			
*APOA1* *(ZPR1-APOA5-A4-C3-A1)* *rs964184*	G	208 (32.0)	223 (26.7)	1.29 (1.03-1.62)	0.03	1.44 (1.11-1.86)	0.01
	G/G	41 (12.6)	37 (8.9)	1.62 (0.99-2.64)	0.05	2.04 (1.11-3.79)	0.02
	G/C	126 (38.8)	149 (35.7)	1.23 (0.90-1.69)	0.18	1.54 (1.04-2.28)	0.03
*LDLR* *rs6511720*	C/C	158 (48.6)	231 (55.4)	1		1	
	T	41 (6.3)	68 (8.0)	0.76 (0.51-1.14)	0.18	0.78 (0.49-1.25)	0.30
	T/T	1 (0.3)	3 (0.7)	NE		NE	
	G/T	39 (11.9)	62 (14.7)	0.78(0.51-1.20)	0.26	0.84 (0.50-1.42)	0.52
	G/G	288 (87.8)	357 (84.6)	1		1	
*APOE* *rs429358*	C	87 (13.5)	72 (8.6)	1.69 (1.21-2.35)	0.002	1.93 (1.30-2.87)	0.001
	C/C	4 (1.3)	3 (0.7)	NE		NE	
	C/T	79 (24.9)	66 (15.8)	1.79 (1.24-2.58)	0.002	2.04 (1.29-3.24)	0.002
	T/T	234 (73.80)	349 (83.5)	1		1	
*APOE* *rs7412*	T	32 (4.9)	63 (7.5)	0.65 (0.42-1.01)	0.05	0.56 (0.34-0.95)	0.03
	T/T	3 (0.9)	1 (0.2)	NE		NE	
	C/T	26 (8.1)	61 (14.5)	0.52 (0.32-0.85)	0.01	0.44 (0.24-0.81)	0.008
	C/C	292 (91.0)	359 (85.3)	1		1	
*APOE-APOC1* *rs4420638*	G	70 (10.7)	57 (6.8)	1.65 (1.15-2.39)	0.01	1.47 (0.93-2.33)	0.10
	G/G	0 (0.0)	0 (0.0)	NE		NE	
	A/G	70 (21.4)	57 (13.5)	1.74 (1.19-2.56)	0.01	1.55 (0.96-2.51)	0.07
	A/A	257 (78.6)	365 (86.5)	1		1	
*IL10* *rs1800872*	T	190 (28.8)	257 (29.9)	0.95 (0.76-1.19)	0.64	1.14 (0.88-1.48)	0.33
	T/T	30 (9.1)	44 (10.2)	0.87 (0.53-1.44)	0.59	1.15 (0.61-2.19)	0.66
	T/G	130 (39.4)	169 (39.3)	0.98 (0.72-1.33)	0.91	1.27 (0.87-1.86)	0.22
	G/G	170 (51.5)	217 (50.5)	1		1	
*IL10* *rs1800896*	C	211 (32.4)	288 (34.4)	0.91 (0.74-1.14)	0.42	0.87 (0.67-1.14)	0.31
	C/C	36 (11.0)	42 (10.0)	0.98 (0.60-1.61)	0.94	0.85 (0.46-1.8)	0.61
	C/T	139 (42.6)	204 (48.7)	0.78 (0.57-1.06)	0.11	1.00 (0.68-1.48)	0.99
	T/T	151 (46.3)	173 (41.3)	1		1	
*TNF* *rs1799964*	C	129 (20.2)	191 (22.9)	0.85 (0.66-1.10)	0.21	0.97 (0.72-1.30)	0.81
	C/C	16 (5.0)	22 (5.3)	0.88 (0.45-1.71)	0.70	1.21 (0.54-2.72)	0.64
	C/T	97 (30.3)	147 (35.2)	0.79 (0.58-1.09)	0.15	0.83 (0.56-1.23)	0.34
	T/T	207 (64.7)	249 (59.6)	1		1	
*IFNG* *rs1861493*	G	152 (23.5)	186 (22.2)	1.08 (0.85-1.38)	0.54	1.00 (0.74-1.35)	0.99
	G/G	9 (2.8)	21 (5.0)	0.61(0.27-1.35)	0.22	0.33(0.12-0.93)	0.04
	G/A	134 (41.5)	144 (34.4)	1.31 (0.97-1.78)	0.08	1.47 (1.00-2.14)	0.05
	A/A	180 (55.7)	254 (60.6)	1		1	
*ABO* *rs579459*	C	130 (19.9)	134 (15.9)	1.32 (1.01-1.72)	0.05	1.28 (0.94-1.74)	0.11
	C/C	10 (3.1)	15 (3.6)	0.98 (0.43-2.21)	0.95	1.27 (0.49-3.26)	0.62
	C/T	110 (33.6)	104 (24.6)	1.55 (1.12-2.13)	0.01	1.32 (0.88-1.97)	0.18
	T/T	207 (63.3)	303 (71.8)	1		1	
*CXCL12* *rs501120*	C	144 (21.9)	239 (27.8)	0.73 (0.57-0.92)	0.01	0.67 (0.50-0.90)	0.008
	C/C	13 (3.9)	35 (8.1)	0.42 (0.22-0.82)	0.01	0.33 (0.14-0.77)	0.01
	C/T	118 (35.9)	169 (39.3)	0.80 (0.59-1.08)	0.14	0.79 (0.54-1.16)	0.23
	T/T	198 (60.2)	226 (52.6)	1		1	
*CXCL12* *rs1746048*	T	138 (21.2)	235 (28.0)	0.69 (0.54-0.88)	0.003	0.67 (0.50-0.89)	0.007
	T/T	12 (3.7)	36 (8.6)	0.37 (0.19-0.73)	0.004	0.32 (0.14-0.75)	0.01
	C/T	114 (35.1)	163 (38.9)	0.77 (0.57-1.05)	0.10	0.81 (0.55-1.19)	0.28
	C/C	199 (61.2)	220 (52.5)	1		1	
*SERPINE1* *rs2227631*	A	229 (34.7)	306 (35.6)	0.96 (0.78-1.19)	0.71	0.96 (0.74-1.23)	0.72
	A/A	43 (13.0)	53 (12.3)	1.00 (0.63-1.58)	0.99	1.03 (0.59-1.81)	0.92
	A/G	143 (43.3)	200 (46.5)	0.88 (0.65-1.20)	0.41	0.74 (0.50-1.09)	0.13
	G/G	144 (43.6)	177 (41.2)	1		1	
*ITGB-3* *rs5918*	C	78 (11.9)	104 (12.1)	0.98 (0.71-1.33)	0.87	0.79 (0.55-1.15)	0.23
	C/C	5 (1.5)	6 (1.4)	1.08 (0.33-3.57)	0.90	0.59 (0.14-2.43)	0.46
	C/T	68 (20.7)	92 (21.4)	0.96 (0.67-1.36)	0.80	0.80 (0.51-1.25)	0.33
	T/T	256 (77.8)	331 (77.2)	1		1	

SNP: single nucleotide polymorphism; OR: odds ratio; CI: confidence interval; NE: non-estimated

^a^ Odds ratios were adjusted by age, gender, education level, European ethnicity, diabetes, and hypertension. For variants related to lipid metabolism, the odds ratios were also adjusted by dyslipidemia.

**Supplementary table 5.** Haplotype distribution in acute coronary syndrome patients and healthy controls

Gen (SNP)	Haplotype	Acute coronary syndrome	Controls	OR (95% IC) Raw	OR (95% IC) Adjusted^a^
*SORT1* (rs646776-rs599839)	*CG*	0.19	0.23	0.76 (0.59-0.98)	0.64 (0.47-0.86)
	*TG*	0.06	0.06	0.86 (0.56-1.31)	0.98 (0.60-1.61)
	*TA*	0.76	0.70	1	1
*LPA* (rs3798220-rs10455872)	*TG*	0.10	0.04	2.89 (1.84-4.52)	2.78 (1.65-4.68)
	*CA*	0.14	0.14	1.08 (0.81-1.45)	1.15 (0.81-1.64)
	*TA*	0.76	0.82	**	**
*APOE* (rs429358-rs7412)	*TT(ε 2)*	0.05	0.08	0.72 (0.46-1.12)	0.63 (0.37-1.05)
	*CC(ε 4)*	0.14	0.09	1.67 (1.18-2.34)	1.86 (1.25-2.78)
	*TC(ε 3)*	0.81	0.84	1	1
*APOE - APOC1* (rs429358- rs7412-rs4420638)	*CCG*	0.07	0.03	2.41 (1.42-4.09)	1.94 (1.04-3.63)
	*TCG*	0.04	0.03	1.22 (0.67-2.22)	1.16 (0.55-2.46)
	*TTA*	0.05	0.07	0.68 (0.43-1.08)	0.60 (0.35-1.03)
	*CCA*	0.07	0.06	1.32 (0.85-2.04)	1.83 (1.01-3.04)
*IL10* (rs1800872-rs1800896)	*TCA*	0.78	0.81	**	**
	*GC*	0.32	0.34	0.87 (0.68-1.11)	0.91 (0.68-1.21)
	*TT*	0.29	0.30	0.90 (0.70-1.15)	1.10 (0.82-1.47)
	*GT*	0.39	0.36	1	1
*CXCL12* (rs501120-rs1746048)	*CT*	0.21	0.28	0.71 (0.55-0.90)	0.66 (0.49-0.89)
	*TC*	0.79	0.72	1	1

SNP: single nucleotide polymorphism; OR: odds ratio; CI: confidence interval

^a^ Odds ratios were adjusted by age, gender, education level, European ethnicity, diabetes, hypertension, and smoking. For the haplotypes of IL10 and CXCL12, the odds ratios were also adjusted by dyslipidemia.
